# Multi-omics of tertiary lymphoid structures in breast cancer: clinical significance and biomarker potential

**DOI:** 10.3389/fimmu.2026.1892848

**Published:** 2026-10-22

**Authors:** Chang’e Chen, Haiping Song

**Affiliations:** Department of Breast and Thyroid Surgery, Union Hospital, Tongji Medical College, Huazhong University of Science and Technology, Wuhan, China

**Keywords:** biomarker, breast cancer, cancer immunotherapy, single-cell RNA sequencing, spatial transcriptomics, tertiary lymphoid structures

## Abstract

**Background:**

Tertiary lymphoid structures (TLSs) are organized immune aggregates that can support local adaptive immune responses within the tumor microenvironment. In breast cancer, particularly triple-negative breast cancer (TNBC), the presence and maturation of TLSs have been associated with prognosis and treatment response; however, heterogeneity in TLS definition, maturation criteria, spatial sampling, and analytical platforms currently limits standardization and routine clinical implementation.

**Main body:**

Conventional histopathology provides structural information but limited molecular resolution, whereas single-cell RNA sequencing loses the spatial context that is central to TLS organization. High-resolution spatial and multi-omics technologies can partly bridge these gaps by characterizing TLS cellular composition, molecular interactions, and tissue architecture *in situ*. This review summarizes the biological features, histopathological classification, multi-omics characterization, clinical associations, and emerging therapeutic strategies related to TLSs in breast cancer, with particular attention to methodological limitations and translational barriers.

**Conclusion:**

More precise characterization of TLSs may inform future biomarker development and therapeutic strategies in breast cancer. Prospective validation, standardized assessment, and demonstration of incremental clinical utility are required before TLS-based approaches can be incorporated into routine clinical decision-making.

## 1 Introduction

Breast cancer (BC) is the most commonly diagnosed malignancy worldwide and remains a leading cause of cancer-related death among women. In 2022, breast cancer accounted for nearly one-quarter of all new cancer cases diagnosed in women (1). Breast cancer is commonly classified into four major molecular subtypes: Luminal A, Luminal B, HER2-enriched, and triple-negative breast cancer (TNBC). Among these, TNBC poses substantial clinical challenges because of its aggressive behavior and limited availability of established molecular targets.

Although breast cancer treatment has evolved from traditional approaches to include neoadjuvant therapy and immune checkpoint inhibitors (ICIs) like pembrolizumab, clinical efficacy remains significantly heterogeneous. Currently, immune checkpoint inhibitors combined with chemotherapy have become the standard treatment in early-stage TNBC (2, 3). However, a substantial number of patients fail to achieve durable responses due to primary or acquired resistance and treatment-related toxicities (4–6). This clinical bottleneck urgently calls for reliable biomarkers to guide patient stratification and reveal tumor immune escape mechanisms.

Within the tumor microenvironment (TME), tertiary lymphoid structures (TLSs) are organized lymphoid aggregates that can support local adaptive immune responses. Their presence, particularly when mature, has been associated with favorable prognosis and immunotherapy response in several tumor types (7–11).

However, the magnitude and independence of these associations vary by cancer type, disease setting, TLS definition, spatial sampling, and assessment method. Conventional histology, single-cell profiling, and spatial omics interrogate different aspects of TLS biology and each has important technical limitations (12–14).

Several recent reviews have discussed the biology and clinical relevance of TLSs in breast cancer. Here, we focus on how histopathology, single-cell analysis, spatial transcriptomics, and multi-omics approaches contribute to TLS characterization, with particular attention to their strengths and limitations. By integrating findings from these complementary approaches, we summarize current understanding of TLS formation and maturation, cellular interactions, and spatial organization in breast cancer, together with their clinical significance and potential therapeutic implications. We also discuss the major challenges that currently limit standardized TLS assessment and the translation of multi-omics findings into clinical practice.

## 2 Biological features and histopathological classification of TLSs in breast cancer

### 2.1 Definition and canonical architecture of TLSs

Under pathological conditions such as chronic inflammation, autoimmune diseases, and cancer, ectopic lymphoid aggregates can develop in non-lymphoid tissues. These structures, known as tertiary lymphoid structures (TLSs), share important structural and functional characteristics with secondary lymphoid organs (SLOs), although they arise in response to local inflammatory cues rather than during embryonic development (8, 9). In the breast tumor microenvironment, persistent inflammatory signaling and immune activation promote the recruitment and spatial organization of lymphoid and stromal components, ultimately giving rise to TLSs.

The formation of TLSs is coordinated by inflammatory, lymphotoxin, chemokine, and stromal programs. Recent mechanistic and spatial studies have implicated CXCL13, CCL19, and CCL21 in the recruitment and organization of lymphocytes, while CCL19-expressing perivascular stromal populations and endothelial acquisition of HEV-like phenotypes may further support TLS organization and maturation (10). In this way, TLSs serve as ectopic sites for local antigen presentation, lymphocyte activation, and adaptive immune coordination.

Canonical TLS architecture typically includes segregated B-cell follicles and T-cell-rich zones together with antigen-presenting, stromal, and vascular elements. More developed TLSs may contain follicular dendritic-cell (FDC) networks, activated dendritic cells, and high endothelial venules (HEVs), which together support lymphocyte recruitment and local adaptive immune coordination (8, 9, 15).

### 2.2 Maturation states of TLSs

The maturation status of TLSs is an important feature associated with their immunological function and clinical relevance. TLS formation is generally considered a dynamic process that progresses from early lymphoid aggregation toward increasingly organized immune microenvironments. Based on structural organization and cellular composition, TLSs are commonly classified into three major maturation states: early TLSs (E-TLSs), primary follicle-like TLSs (PFL-TLSs), and secondary follicle-like TLSs (SFL-TLSs) (16).

E-TLSs represent the earliest and least organized stage of TLS development, with lymphocytic aggregates but limited follicular organization or germinal-center activity. PFL-TLSs show clearer B- and T-cell compartmentalization and FDC development, whereas SFL-TLSs contain germinal-center features consistent with greater functional maturation (15, 16).

The most mature stage, SFL-TLSs, is characterized by germinal-center (GC) formation and is often regarded as the closest ectopic counterpart of an organized secondary lymphoid follicle. Mature TLSs may contain a B-cell core enriched in BCL6+ cells, an interconnected CD21+CD23+ FDC network, and surrounding PNAd+ HEVs. These features can support B-cell affinity maturation, class switching, and the generation of plasma cells and memory B cells. Studies integrating spatial transcriptomics and B-cell receptor (BCR) repertoire analysis further suggest that TLS maturation is accompanied by progressive molecular specialization, consistent with a transition from structural assembly toward greater functional competence (17).

Because germinal-center formation and molecular specialization are linked to greater functional competence, many clinical studies now distinguish mature from immature TLSs rather than using presence alone (11, 16).

### 2.3 Histopathological identification and current limitations

Histopathological evaluation remains the most widely used approach for identifying TLSs in tumor tissues. On routine hematoxylin and eosin (H&E) staining, TLSs are initially recognized as dense lymphoid aggregates within or adjacent to the tumor. However, morphology alone is often insufficient to determine their maturation status or functional properties. Therefore, immunohistochemistry (IHC) and multiplex immunofluorescence (mIF) are frequently used to refine TLS classification by visualizing specific cellular compartments and structural markers, such as CD20+ B-cell areas, CD3+ T-cell zones, CD21+ or CD23+ FDC networks, and PNAd+ HEVs (12).

These pathology-based approaches have significantly improved our ability to distinguish immature from mature TLSs and to evaluate their spatial relationship with tumor cells and stromal regions. Nevertheless, several important limitations remain. First, the histopathological criteria used to define TLSs are not yet standardized across studies. Different groups vary in whether they require clear B/T cell compartmentalization, FDC networks, HEVs, or germinal center formation for TLS classification. This inconsistency complicates cross-study comparisons and limits the reproducibility of TLS-based biomarker analyses. One commonly used operational framework includes lymphoid aggregates containing more than 50 cells and further classifies TLSs as mature when CD23+ dendritic-appearing follicular dendritic cells and/or germinal-center features are identified (11). Importantly, the >50-cell threshold and the markers used to define maturation are study-specific operational criteria rather than universally accepted standards. Second, conventional morphology-based assessment provides only a static snapshot of TLS architecture and cannot fully capture the cellular heterogeneity, activation states, or molecular interactions that determine TLS function. A morphologically recognizable lymphoid aggregate may therefore not necessarily correspond to a functionally competent TLS.

Recent advances in single-cell RNA sequencing and spatial transcriptomics have helped address some of these limitations by resolving TLS-associated cellular diversity, transcriptional programs, and spatial organization at higher resolution. These studies suggest that TLS abundance, cellular composition, localization, and maturation state may be associated with clinical outcomes, particularly in immunotherapy settings (18). Nevertheless, routine clinical implementation remains constrained by cost, technical complexity, sampling variability, and the lack of harmonized analytical standards.

Artificial intelligence and deep-learning approaches are increasingly being evaluated as tools to improve the objectivity and reproducibility of TLS assessment in digital pathology. These methods can assist with automated identification, annotation, and quantification of TLSs across whole-slide images and may support future standardized evaluation frameworks (13). However, robust external validation across platforms, cohorts, specimen types, and breast cancer subtypes is required before such tools can be adopted in routine practice.

### 2.4 Transcriptomic signatures as surrogate markers of TLS: applications and controversies in prognostic models

Transcriptomic gene signatures are increasingly used as molecular surrogates of TLS-associated immune activity and as components of prognostic or predictive models. Their main advantages are quantitative scoring and compatibility with bulk or spatial transcriptomic datasets and, depending on the platform, archival tumor specimens. A range of such signatures has been reported across tumor types (14, 19–24) (**Table 1**). However, these signatures infer TLS-related biology from gene expression and do not directly demonstrate the presence, location, or maturation of an organized TLS.

**Table 1:** Representative transcriptomic signatures associated with tertiary lymphoid structures across cancer types.

Signature	Gene composition	Original cancer	Reported prognostic, predictive, or analytical performance	Ref.
12-chemokine TLS gene-expression signature (12-CK GES)	CCL2, CCL3, CCL4, CCL5, CCL8, CCL18, CCL19, CCL21, CXCL9, CXCL10, CXCL11, CXCL13	Colorectal carcinoma (CRC)	Identified TL-ELNs/TLS and was associated with better patient outcome in CRC.	(20)
8-gene Tfh signature	CXCL13, CD200, FBLN7, ICOS, SGPP2, SH2D1A, TIGIT, PDCD1	Breast cancer	Associated with better DFS and higher pCR to neoadjuvant chemotherapy, particularly in HER2+ breast cancer.	(21)
29-gene TLS imprint signature	IGHA1, IGHG1, IGHG2, IGHG3, IGHG4, IGHGP, IGHM, IGKC, IGLC1, IGLC2, IGLC3, JCHAIN, CD52, CD79A, FCRL5, MZB1, SSR4, XBP1, TRBC2, IL7R, CXCL12, LUM, C1QA, C7, APOE, PTLP, PTGDS, PIM2, DERL3	Clear cell renal cell carcinoma (ccRCC)	Reported TLS-region classification AUCs of 0.89-0.97 in discovery and 0.77-0.94 in frozen validation cohorts.	(17)
single-cell & spatial TLS (ssTLS) signature	CD3D, CD3E, CD19, MS4A1, CD79A, CXCL13, CXCR5, CXCL16, CCL19, CCL22, ACKR1, SELE	Gastric cancer	Reported anti-PD-1 response discrimination (AUC = 0.795) and associations with prolonged PFS and OS.	(22)
30-gene spatial transcriptomics-derived TLS signature (TLS ST signature)	CD79A, CD79B, TNFRSF13C, BLK, CD22, CD37, MS4A1, NIBAN3, CD19, IKZF3, LINC00926, CXCR5, LTB, SELL, CCL19, POU2AF1, CXCL13, FCRLA, VPREB3, FCMR, AL928768.3, RASGRP2, TCF7, RIPOR2, RAC2, IL16, CCR7, CD52, TCL1A, ATP2A3	Triple-negative breast cancer	Higher scores were associated with improved DRFS across ST-TNBC, METABRIC, and SCAN-B cohorts (combined n=944). Higher expression was also associated with pCR in pembrolizumab-treated early TNBC in I-SPY2 and with PFS/RECIST response across independent ICI-treated metastatic cancer cohorts.	(14)
TLS9/Cabrita 9-gene signature	CD79B, CD1D, CCR6, LAT, SKAP1, CETP, EIF1AY, RBP5, PTGDS	Melanoma	Originally associated with favorable outcome and ICB response in melanoma.	(19)
Common TLS-associated hallmark genes	CCL19, CCL21, CXCL13, CCR7, CXCR5, SELL, LAMP3	Various cancers	Used as molecular indicators of TLS-associated immune activation; not established as a standalone prognostic or predictive signature.	(15)
4-gene TLS signature	PAX5, TCL1A, TNFRSF13C, CD79A	Cholangiocarcinoma (CCA)	Identified TLSs in the original CCA analysis but was not significantly associated with patient survival.	(23)
TLS-50 signature	FDCSP, CR2, CXCL13, LTF, CD52, MS4A1, CCL19, LINC00926, LTB, CORO1A, CD79B, TXNIP, CD19, LIMD2, CD37, ARHGAP45, BLK, TMC8, CCL21, PTPN6, ATP2A3, IGHM, SPIB, TMSB4X, CXCR4, NCF1, CD79A, ARHGAP9, DEF6, EVL, TBC1D10C, RASAL3, INPP5D, RNASET2, RASGRP2, TNFRSF13C, RAC2, CD22, ARHGEF1, AC103591.3, TRAF3IP3, HLA-DQB1, CD53, ARHGAP4, TRBC2, POU2AF1, TRAF5, OGA, FCRL3, HLA-DQA1	Hepatocellular carcinoma (HCC)	Identified TLS regions in the original HCC analysis; a high TLS-50 score was associated with better survival (P = 0.012).	(24)

For example, a 30-gene TLS-related signature was derived using spatial transcriptomic analysis of triple-negative breast cancer (14). The TLS9 signature (CD79B, CD1D, CCR6, LAT, SKAP1, CETP, EIF1AY, RBP5, and PTGDS) was originally developed in melanoma and has subsequently shown prognostic associations in breast cancer (19). Reported performance varies by signature, cancer type, cohort, and endpoint; therefore, direct comparisons between signatures should be made cautiously.

Several limitations should be considered when interpreting these signatures. Not all gene sets have been spatially validated against structurally intact TLSs, particularly mature SFL-TLSs; TLS-associated genes such as CXCL13 may also be expressed by dispersed infiltrating lymphocytes; and transcriptomic scores generally cannot distinguish germinal-center-positive functional TLSs from non-functional lymphoid aggregates or capture three-dimensional TLS architecture (15, 23, 24).

Accordingly, transcriptomic TLS scores should currently be regarded as molecular surrogates rather than direct substitutes for histopathological or spatially validated TLS assessment. Integration with spatially resolved methods may improve biological specificity, but standardized analytical pipelines and prospective clinical validation are still required before these scores can be used for routine patient stratification.

## 3 Why multi-omics is needed: from morphological observation to spatially resolved molecular characterization

The limitations of morphology-only and dissociated-cell approaches outlined above motivate complementary imaging, single-cell, and spatial methods, each with different trade-offs in resolution, molecular breadth, throughput, and preservation of tissue context (25, 26).

### 3.1 Multiplex imaging technologies

Multiplex imaging can delineate the cellular and structural organization of TLSs *in situ*, including B- and T-cell compartmentalization, plasma-cell-rich regions, FDC networks, and HEV-associated features. By preserving tissue architecture while measuring multiple markers simultaneously, these approaches complement conventional H&E/IHC and help link morphology with molecular phenotype. Interpretation nevertheless depends on marker selection, image segmentation, spatial resolution, and platform-specific analytical pipelines (27).

### 3.2 Single-cell transcriptomics

In TLS research, the main contribution of scRNA-seq is its ability to define, at single-cell resolution, the specific cell phenotypes involved in TLS formation and function (**Figure 1**, left). Through high-throughput gene expression profiling, researchers have identified key subsets residing in TLS, such as CXCL13^+^ follicular helper T cells (28, 29). LAMP3^+^ mature DCs (30), and B cells with distinct functional states (31, 32). Pseudotime analysis has also helped reconstruct the differentiation trajectories of these cells within the tumor microenvironment. Furthermore, receptor–ligand inference algorithms have uncovered complex intercellular communication networks driving TLS formation, such as chemokine interactions between fibroblasts and lymphocytes (33).

**Figure 1:**
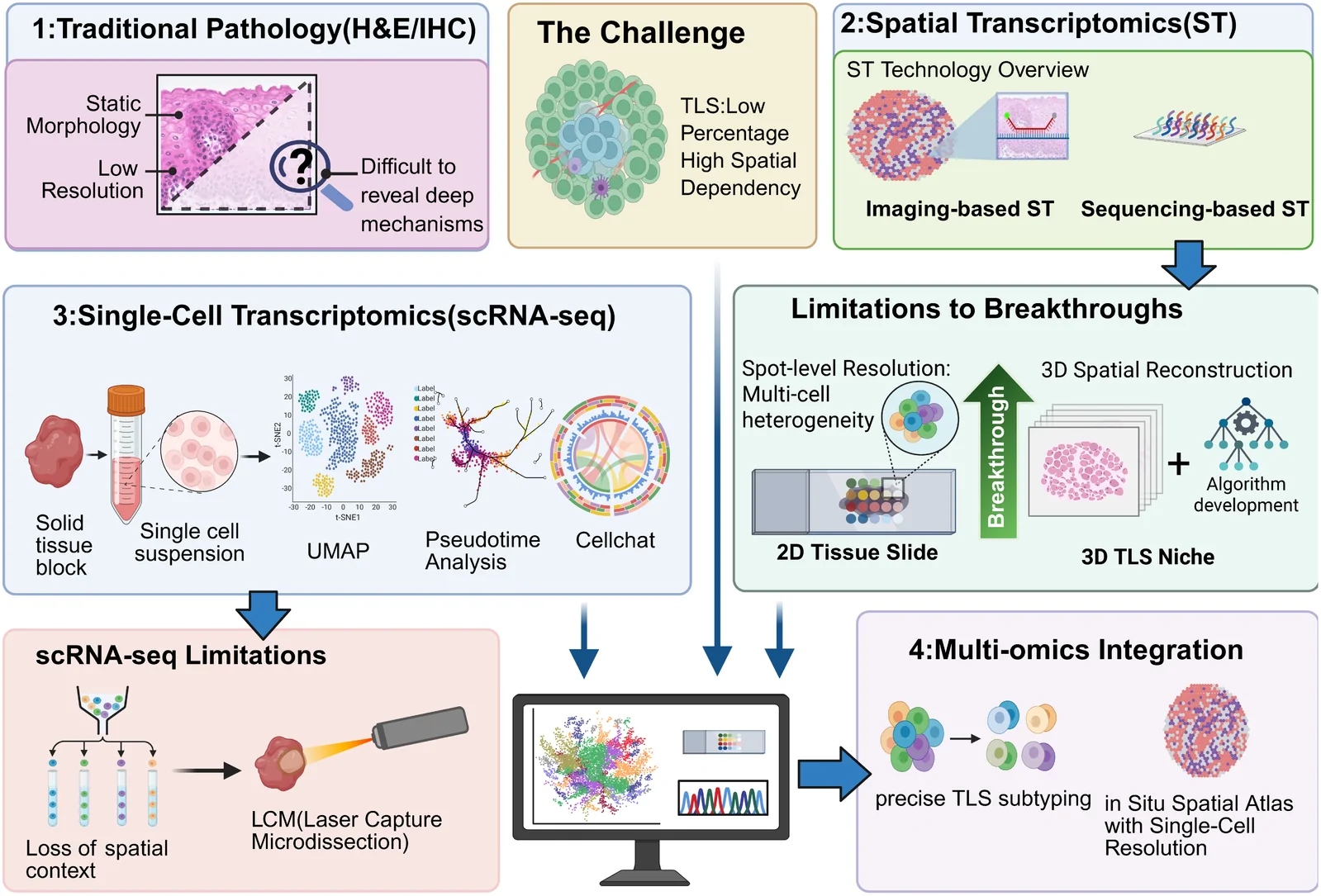
Multimodal integration strategies for analyzing tertiary lymphoid structures (TLS). (Top) TLS research challenges and complementary analytical strategies. (Left) Histopathology and scRNA-seq: Conventional H&E and immunohistochemistry primarily provide morphological context, whereas scRNA-seq can resolve cell states, trajectories, and inferred intercellular communication. Tissue dissociation, however, removes native spatial relationships. (Right) Spatial transcriptomics (ST): Imaging-based and sequencing-based approaches generate *in situ* gene-expression maps with different trade-offs in resolution and transcriptome breadth. Conventional array-based ST can mix signals from multiple cells, and three-dimensional reconstruction remains technically challenging. (Bottom) Multimodal integration: Computational frameworks can integrate scRNA-seq, spatial transcriptomics, and tissue imaging to improve characterization of TLS composition and organization, although platform harmonization and validation remain necessary.

However, scRNA-seq faces a fundamental technical challenge when applied to TLS. The sample preparation process requires complete enzymatic digestion of tissue. Since TLS is a micro-organ whose function depends heavily on its spatial architecture, and its cells account for only a tiny fraction of the bulk tumor, performing scRNA-seq on whole tumor samples often introduces background noise. It becomes difficult to determine whether the sequenced lymphocytes originated from structurally organized TLS or were merely scattered throughout the stroma. To address this “dilution effect,” early researchers used laser capture microdissection (LCM) to isolate TLS regions from frozen tissue before performing transcriptomic analysis or qRT-PCR. This approach enriched TLS-specific signals to some extent but remained limited in both throughput and resolution (34).

### 3.3 Spatial transcriptomics and spatial multi-omics

Spatial transcriptomics (ST) has become an important research tool for studying TLS organization *in situ* (**Figure 1**, right). Current approaches can be broadly divided into imaging-based and sequencing-based methods. Imaging-based methods, including *in situ* hybridization and *in situ* sequencing platforms such as MERFISH, 10x Xenium, and CosMx, enable highly resolved visualization of selected transcripts and are well suited to analyses of subcellular localization, low-abundance targets, and fine cellular states. Sequencing-based methods capture transcripts using spatial barcodes and can provide broader transcriptome coverage; examples include array-based platforms such as Visium and Stereo-seq and region-of-interest approaches. These technologies offer complementary trade-offs in resolution, transcriptome breadth, throughput, cost, and tissue compatibility (25).

That said, most current ST platforms have not yet achieved true single-cell resolution. Each detection spot typically contains several to dozens of cells, masking some degree of cellular heterogeneity within the spot. This has become a key limitation when analyzing fine TLS structures. For example, a standard Visium spot is about 55 μm in diameter and often contains a mix of cell types. This makes it difficult to define precise boundaries between different functional compartments within TLS, such as the germinal center and T-cell zone (35). Visium HD improves spatial resolution by using 2 μm × 2 μm square barcode units as native detection elements, though these are often merged into 8 μm × 8 μm grids in practice (36). While this significantly enhances resolution, it still falls short of true single-cell precision. Because the technology relies on *in situ* mRNA capture rather than physical isolation of single cells, transcripts from densely packed TLS cells can diffuse into adjacent areas, mixing signals from different cells. Computational cell segmentation is then required, which inevitably introduces errors (26).

### 3.4 Integrative, three-dimensional, and AI-assisted approaches

Integrative spatial profiling can connect morphology, cellular composition, and molecular state within the same TLS. (**Figure 1**, Bottom)In a large glioma study, GeoMx digital spatial profiling, Xenium, whole-slide multiplex immunofluorescence, and high-plex immunofluorescence were used to define transcriptionally and phenotypically distinct TLS states, including T-cell-low, B/T-cell-enriched (B:T-TLS), and plasma-cell-enriched (PC-TLS) states (27). This example illustrates the value of multimodal profiling for resolving TLS heterogeneity, while cross-tumor generalizability remains to be established.

Three-dimensional reconstruction of serial tissue sections combined with computational analysis has further suggested that TLSs that appear discrete in two-dimensional sections may form interconnected network-like structures (TLSNs) in three-dimensional space (37). This observation provides a useful framework for considering TLS spatial organization beyond isolated lymphoid aggregates, although the generalizability and functional significance of TLSNs require further investigation.

Deep-learning tools such as GARDEN have also been developed to assist in identifying small or low-abundance TLSs in spatial datasets and have reported improved detection performance in the study settings in which they were evaluated (38). However, the high cost of spatial transcriptomics, platform-specific analytical requirements, and the complexity of integrating H&E, immunofluorescence, and single-cell data remain substantial barriers to routine clinical translation.

Overall, these approaches provide complementary rather than interchangeable information for TLS characterization. Multiplex imaging preserves tissue architecture and enables direct assessment of cellular organization but is limited by predefined marker panels. scRNA-seq provides high-resolution characterization of cellular states and interactions but loses native spatial context. Spatial transcriptomic approaches partially bridge this gap by linking molecular programs to tissue localization, although their resolution, molecular coverage, cost, and analytical requirements vary across platforms. Integrative analysis may therefore provide the most comprehensive characterization of TLSs, but at the expense of greater technical and computational complexity.

## 4 Cellular atlas and functional crosstalk within TLSs

TLSs are organized immune niches within the tumor microenvironment whose biological activity depends on coordinated interactions among lymphoid, myeloid, stromal, and vascular cell populations. Multi-omics studies have mapped this composition at high resolution and generated mechanistic hypotheses about ligand-receptor interactions and signaling networks that may support local adaptive immune responses (14, 17, 33, 39) (**Figure 2**).

**Figure 2:**
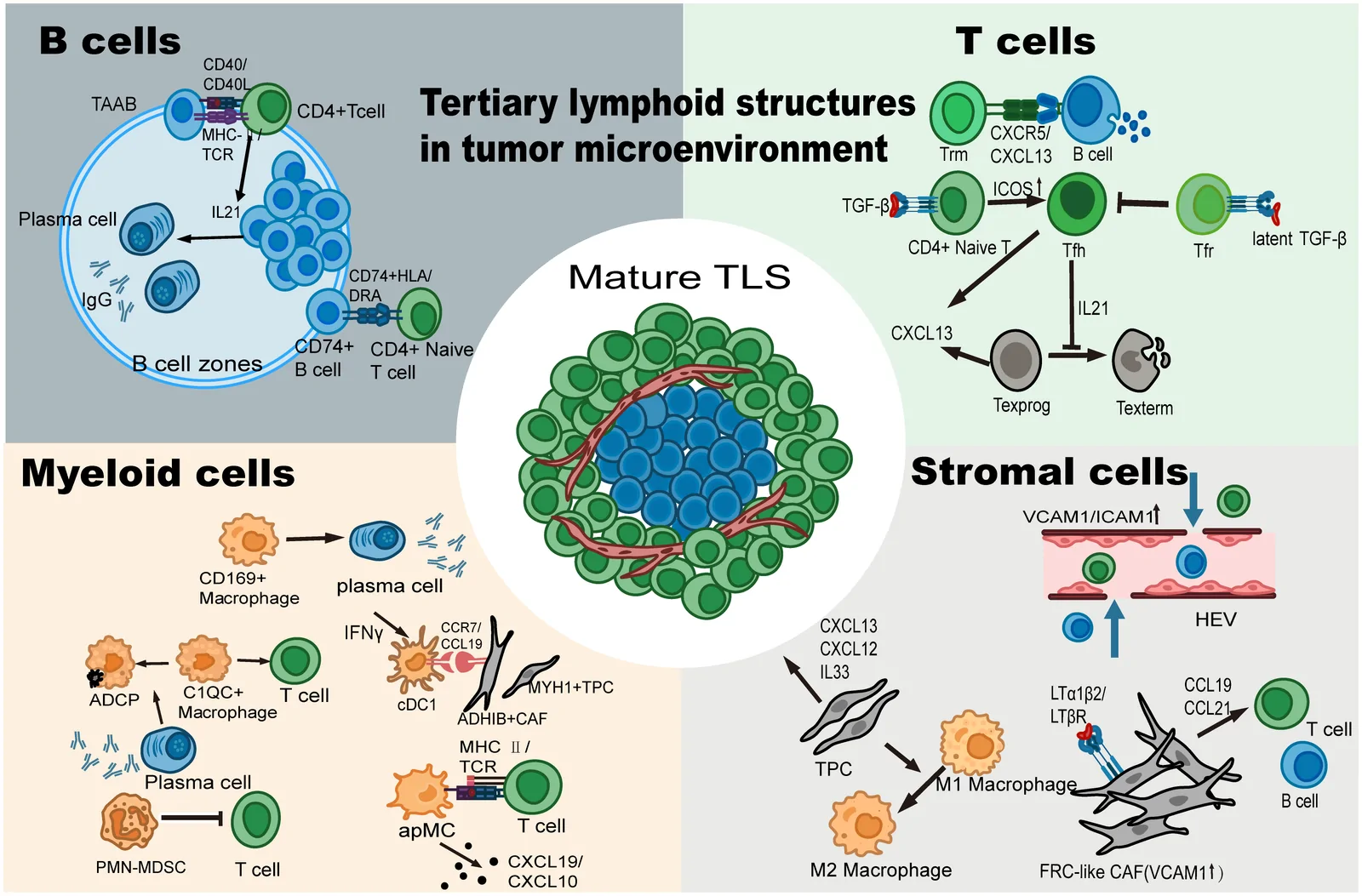
Cell atlas and functional crosstalk within TLS from a multi-omics perspective. (Center) A mature TLS with organized B-cell and T-cell compartments and associated vascular structures. (Upper left) B-cell programs include antigen presentation, T-cell interaction, plasma-cell differentiation, and antibody production. (Upper right) T-cell programs include Trm–B-cell interactions, Tfh/Tfr regulation, and exhausted T-cell states. (Lower left) Myeloid-cell interactions involve dendritic cells, macrophages, antigen-presenting mast cells, and PMN-MDSCs, with both immune-supportive and suppressive functions. (Lower right) Stromal and vascular components, including HEVs, pericytes, and FRC-like CAFs, contribute to lymphocyte recruitment and TLS organization.

### 4.1 B-cell lineage and humoral immune programs

Single-cell RNA sequencing studies have identified a spectrum of B-cell states within TLSs, ranging from naive B cells to plasmablasts and plasma cells, together with somatic hypermutation and clonal expansion. These observations support the concept that at least a subset of mature TLSs can provide local sites for B-cell activation and antibody-associated immune responses (17).Spatial transcriptomic analyses further indicate that germinal-center B cells and plasmablasts can be enriched in TLS cores. CD74+ B cells have also been described as antigen-presenting populations with differentiation potential and may interact with naive T cells through HLA class II-associated pathways (40). In the breast cancer microenvironment, FCRL4+ memory B cells have been described as tumor-associated atypical B cells (TAABs) that spatially associate with CXCL13+CD4+ T cells. Proposed reciprocal signaling through antigen presentation, costimulatory pathways, and IL-21 may support TAAB activation and plasma-cell differentiation (41) (**Figures 3A–F**).

**Figure 3:**
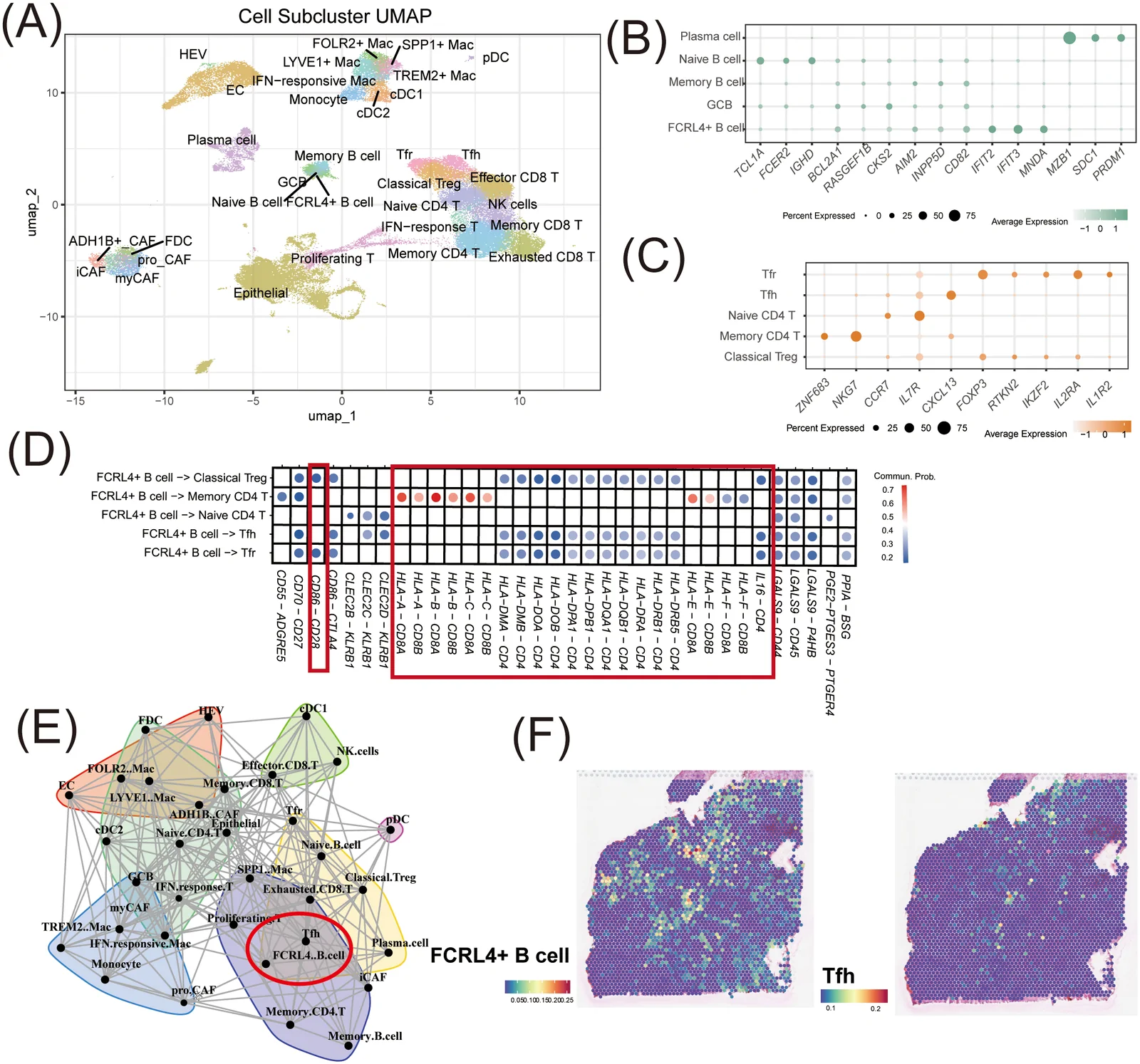
Cellular communication and spatial colocalization between TAABs and T cells in the breast cancer immune microenvironment. All panels were generated by the authors through secondary analysis of previously published public datasets; no new patient specimens or sequencing data were generated. **(A–C)** Immune-cell subclustering of all 26 breast cancer samples from the scRNA-seq dataset GSE176078 (42) using Seurat with quality control, doublet removal, cell-cycle regression, Harmony correction, and dimensionality reduction. **(D)** CellChat (v2.1.2) analysis of communication between tumor-associated atypical B cells (TAABs) and T-cell subsets. **(E, F)** Spatial colocalization of TAABs and Tfh cells from the TNBC spatial transcriptomic dataset GSE252176 (43), using RCTD-derived cell-type proportions and MistyR.

### 4.2 T-cell subsets and local adaptive immunity

Different T cell subsets maintain immune homeostasis within TLS through both synergistic and antagonistic interactions, thereby influencing the direction and intensity of antitumor immune responses. In mature TLS, CD8^+^ tissue-resident memory T (Trm) cells are significantly enriched (44). They recruit activated B cells via the CXCL13-CXCR5 signaling axis and upregulate granzyme B expression to enhance their cytotoxic activity (45). Notably, B cells within TLS can reciprocally enhance the effector function of these Trm cells via the LTα/TNFR2-mTOR-glycolytic metabolic axis, forming a positive feedback loop. Beyond CD8^+^ Trm cells, specific CD4^+^ T cell subsets also contribute to TLS formation. For instance, central memory-like CD4^+^ T cells that highly express lymphotoxin β (LTβ) activate MMP2^+^ fibroblasts and FOLR2^+^/CCL4^+^ macrophages via the LTβ-LTβR axis, promoting TLS development (46). Within functional TLS, Tfh cells serve as core drivers of the germinal center response. TGF-β suppresses SATB1 expression in CD4^+^ T cells through SMAD3 signaling, thereby relieving transcriptional repression of ICOS and promoting Tfh differentiation (47). Tfh cells support B cells in undergoing somatic hypermutation of the BCR, enhancing antibody affinity (17). Notably, a subset of functional Tfh cells with a PD-1^hi^ICOS^int^ phenotype also secretes IFN-γ, which boosts CD8^+^ T cell cytotoxicity (48).

In contrast, follicular regulatory T (Tfr) cells (phenotype: CD25^+^CXCR5^+^GARP^+^FOXP3^+^) antagonize Tfh function. They bind and activate latent TGF-β via GARP, suppressing the B cell helper functions and cytokine secretion of Tfh cells (48). The ratio of functional Tfh to Tfr cells within TLS reflects a dynamic balance: in active TLS, this ratio increases, accompanied by germinal center formation, CD8^+^ T cell activation, and high expression of Th1-type genes. This indicates concurrent activation of both humoral and cytotoxic immunity. Clinical studies suggest that this ratio has been associated with favorable prognosis in HER2^+^ and triple-negative breast cancer (48).

T cell exhaustion is also closely linked to TLS formation. Exhausted T cells (Tex) are characterized by reduced cytokine secretion and upregulated expression of inhibitory receptors (PD-1, CTLA-4, LAG3), the immunosuppressive enzyme CD39, and the chemokine CXCL13. Among these, progenitor exhausted T cells (Texprog) are enriched in TLS and lymph nodes. Importantly, CXCL13, a key chemokine for initiating TLS formation, is primarily produced by CD8^+^ exhausted T cells and Tfh cells. Within TLS, the abundance of CD8^+^ Tex cells significantly correlates with and co-localizes with Tfh cells: CD8^+^ Tex cells appear to play an initiating role early in TLS formation, while Tfh cells amplify the chemokine signal (49). Concurrently, Tfh cells, via an IL-21-dependent mechanism, help prevent Texprog cells from differentiating into terminal exhausted T cells (Texterm), thereby preserving a functional T cell reservoir (50).

### 4.3 Myeloid cells and antigen presentation niches

The functional remodeling of myeloid cells is intrinsically linked to the immune activation status of TLS. Following lymphocyte activation, myeloid cells secrete chemokines that provide the structural foundation for TLS formation. In mature TLS (mTLS), these cells drive type 1 interferon receptor signaling, leading to increased tumor-reactive T-cell scores and the upregulation of cytotoxic molecules such as IFNG and GZMA (51). This suggests that myeloid regulation spans the entire TLS-mediated anti-tumor process. TLS-positive tumors are generally associated with a more immunologically active microenvironment, although the composition of myeloid populations varies across tumor types. In HCC, TLS maturation and the balance of N1/N2 tumor-associated neutrophils have been associated with clinical outcome (52), while experimental studies have independently shown that TGF-β signaling promotes a protumor TAN phenotype (39).

Regarding specific subsets, mature DCs primarily localize to the T-cell zone or T-B border within TLS, mirroring lymph node architecture. High expression of HLA-DR and CD86 allows these DCs to efficiently present tumor antigens via MHC-I/II and activate cytotoxic T cells; notably, their density remains independent of germinal center formation. Macrophage-derived pro-inflammatory cytokines provide the necessary inflammatory milieu for DC maturation. IFN-γ drives the maturation of cDC1s, which then home to the intra-tumoral lymphoid area via CCR7-mediated migration—a core step for TLS formation and maintenance (53, 54). During tumor progression, local cDC1s utilize the CCR7-CCL19 axis to migrate toward stromal hubs enriched with ligands (e.g., ADH1B+ CAFs and MYH11+ pericytes), ensuring TLS structural and functional stability.

Macrophages act as central effectors in TLS-mediated regulation. In TLS-positive tumors, C1QC+ macrophages accumulate and adopt an M1-like phenotype (high IL-12, iNOS), recruiting and activating T cells. Additionally, IGHG1/IGHG3 antibodies secreted by TLS plasma cells enhance C1QC+ macrophage phagocytosis via antibody-dependent cellular phagocytosis (ADCP), establishing a synergistic “TLS-plasma cell-macrophage” anti-tumor axis. In contrast, macrophages in TLS-negative samples typically exhibit an immunosuppressive M2 phenotype (high CD163, IL-10), while neutrophils in non-TLS regions suppress T-cell function via neutrophil extracellular traps (51, 55). Moreover, CD169+ resident macrophages co-localize with T and B cells within TLS. Although they inhibit naive B-cell proliferation, they promote IgG and IL-6 secretion by activated B cells, suggesting a role in germinal center regulation (56).

Other myeloid subsets also contribute to this regulatory network. In TNBC, antigen-presenting mast cells (apMCs) within TLS express high levels of CD74. These cells directly activate T-cell TCR signaling via MHC-I/II presentation, compensating for potential deficits in DC function. Furthermore, apMCs secrete chemokines such as CXCL9 and CXCL10 to recruit peripheral T and NK cells, enhancing local immune infiltration (57).

Conversely, polymorphonuclear myeloid-derived suppressor cells (PMN-MDSCs) primarily exert immunosuppressive effects. High levels of PMN-MDSCs in immature TLS (containing primary follicles) inhibit T-cell proliferation and IFN-γ production, impeding the transition to mature, germinal center-positive TLS (18).

Additionally, ferroptotic PMN-MDSCs release lipid peroxidation products that directly suppress T cells, amplifying immunosuppression. Oxidized lipids from these cells also transfer to DCs, impairing antigen cross-presentation and indirectly blocking T-cell activation. Thus, PMN-MDSCs establish a systemic immunosuppressive network through a dual mechanism of arresting TLS maturation and interfering with cellular function (58, 59).

### 4.4 Stromal and vascular components supporting TLS organization

Stromal cells form the physical scaffold of TLS and regulate immune cell trafficking. HEVs serve as the main entry points for lymphocyte infiltration (60). They not only express VCAM1 and ICAM1 to mediate adhesion but also recruit CXCL13^+^ T cells through the CXCL13-ACKR1 pathway, which in turn promotes B cell homing (22). In nasopharyngeal carcinoma, for instance, interferon-responsive HEVs (IFN-HEVs) located at the TLS margin highly express CXCL9/10. Through the CXCL9-CXCR3 axis, they specifically recruit CD4^+^ T cells, and this has been linked to better patient outcomes (61).

Cancer-associated fibroblasts (CAFs) show functional heterogeneity within TLS. While some CAF subsets secrete IL-6 and TGF-β to inhibit B cell activation (62), a distinct population known as fibroblastic reticular cell-like CAFs (FRC-like CAFs) plays a positive role. These cells originate from resident fibroblast precursors and are mainly found in immunoinflamed tumors. They are enriched in the non-canonical NF-κB pathway and are activated by LTα1β2-LTβR signaling. FRC-like CAFs help maintain TLS structure by secreting CCL19/CCL21 and provide costimulatory signals through VCAM1, promoting the infiltration and activation of Tfh cells and B cells (63, 64).

Tumor pericytes (TPCs), in contrast, generally exhibit pro-tumor characteristics. For example, they may secrete IL-33 to recruit MDSCs or induce M2 macrophage polarization, contributing to an immunosuppressive microenvironment (65, 66).

## 5 Clinical significance of TLSs in breast cancer

For clarity, prognostic and predictive associations are distinguished throughout this section. Prognostic value refers to associations between TLS features and clinical outcomes irrespective of a specific treatment, whereas predictive value refers to associations between pretreatment TLS features and response or benefit from a defined therapy.

### 5.1 Prognostic value across breast cancer subtypes

TLS frequency and maturation vary across breast cancer subtypes. Current evidence suggests that mature TLSs are more frequently observed in TNBC and HER2-positive tumors than in luminal tumors, and TNBC has been the most extensively studied subtype in relation to TLS organization and immunotherapy-associated immune phenotypes (67). However, reported frequencies and clinical associations depend on specimen type, spatial sampling, TLS definition, and maturation criteria.

TLS presence, abundance, and maturation have been associated with clinicopathological features and survival outcomes in breast cancer (67–71). These findings support continued evaluation of mature TLSs as candidate prognostic biomarkers; however, their independent value beyond tumor subtype, stage, stromal TILs, and other established clinicopathological variables requires prospective validation.

### 5.2 Treatment-response associations and candidate predictive value

Beyond prognosis, TLS-associated features have been investigated in relation to systemic treatment response. Single-cell and spatial profiling of TNBC has identified distinct immune-response trajectories during pembrolizumab-based therapy. Some responding tumors show evidence of pre-existing TLS-associated immunity, whereas another responder subset develops more pronounced immune activation after the addition of radiotherapy. These observations suggest that baseline immune organization may help characterize response phenotypes, but TLS status alone is insufficient for treatment selection (72, 73).

The effect of neoadjuvant chemotherapy (NAC) on TLSs remains uncertain. Some studies have reported increased TLS formation or maturation after NAC (69, 74), whereas the magnitude and clinical significance of treatment-induced changes are not yet established. Such treatment-induced changes should be considered pharmacodynamic observations rather than evidence of pretreatment predictive value. Longitudinal studies using standardized sampling and TLS criteria are needed to determine how chemotherapy and radiotherapy remodel TLSs.

Across tumor types, TLS presence, maturation, and treatment-associated changes have been reported in relation to systemic therapy response. In potentially resectable hepatocellular carcinoma treated with nivolumab plus ipilimumab, increased TLS-related signatures and treatment-associated TLS formation were observed in responders. In melanoma patients receiving TIL therapy, responders showed greater abundance of class-switched B cells and higher TLS scores than non-responders (75, 76). TLS enrichment has also been associated with pembrolizumab response in advanced soft-tissue sarcoma (77), and TLS-rich inflammatory phenotypes have been described among nivolumab responders in mesothelioma (78).

Although these observations suggest that TLS-associated immune organization may track therapeutic responsiveness in several tumor types, direct extrapolation to breast cancer is limited. Breast cancer-specific prospective studies are needed to determine whether TLS assessment provides predictive information beyond established biomarkers and clinicopathological factors.

### 5.3 Spatial context matters: intratumoral, peritumoral TLSs

The clinical associations of TLSs may depend not only on their presence and maturity but also on their spatial distribution. In breast cancer, peritumoral and intratumoral TLS patterns have been associated with clinicopathological features and treatment outcomes (68–70). These observations suggest that spatial context may influence TLS-associated immune activity, but definitions of TLS location and sampling strategies vary across studies.

The local tumor microenvironment may also influence TLS distribution. Three-dimensional imaging studies have reported spatial exclusion between endogenous microbiota colonization sites and TLS clusters in breast cancer tissue, raising the possibility that local microbial, metabolic, or immunoregulatory factors affect TLS organization (79). These findings remain mechanistic and require validation in larger clinical cohorts, but they illustrate why spatially resolved evaluation may provide information beyond simple presence-or-absence scoring.

Taken together, subtype, maturation state, treatment-associated dynamics, and spatial distribution are all candidate dimensions for future TLS-informed prognostic models (67–72) (**Figure 4**). A clinically useful framework would need to define these variables reproducibly and establish whether they add information beyond conventional pathology and existing immune biomarkers.

**Figure 4:**
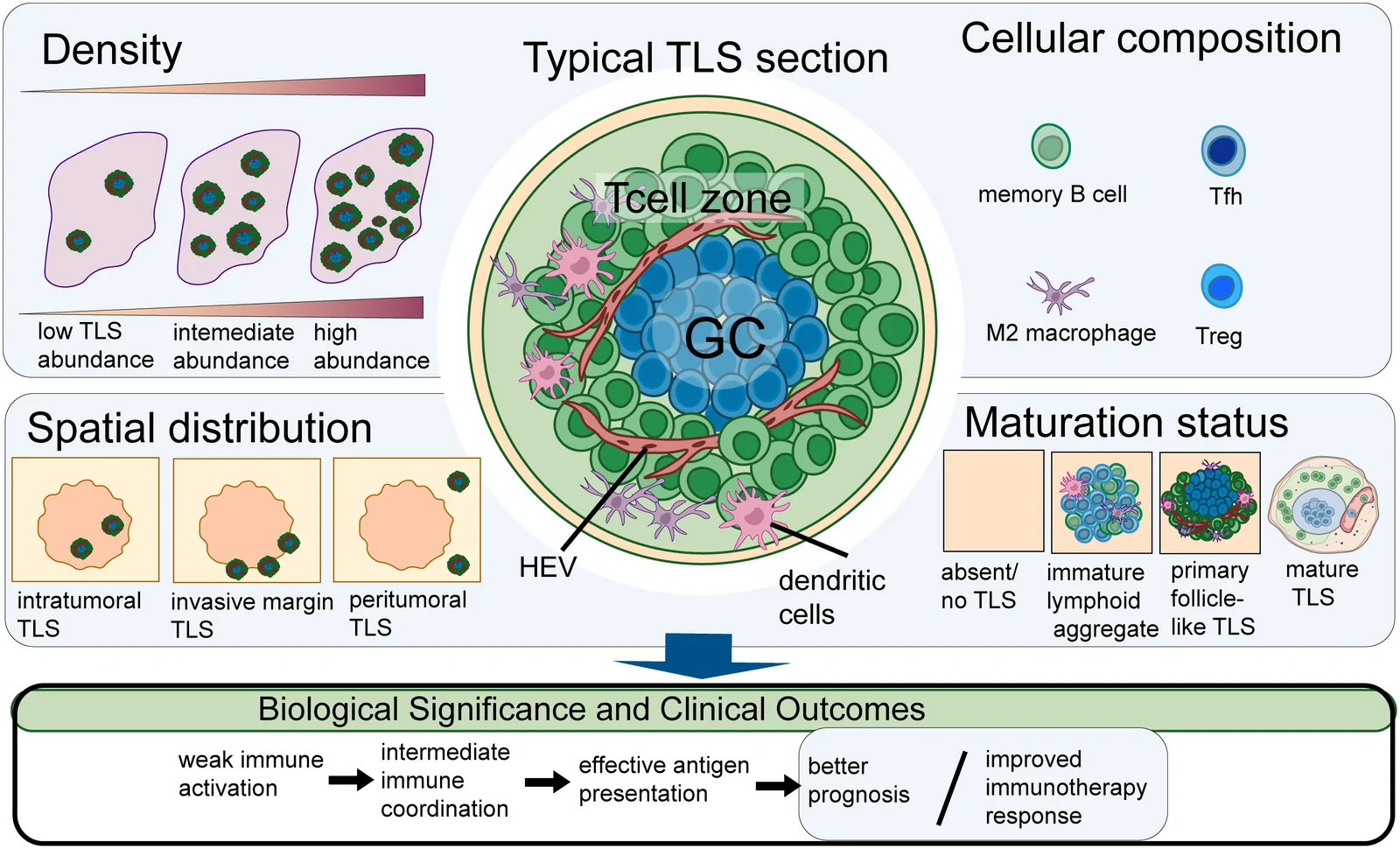
Multidimensional features of tertiary lymphoid structures (TLSs) associated with clinical outcomes in breast cancer. The clinical relevance of TLSs may depend on several key dimensions. This figure illustrates four core attributes for evaluating TLS-mediated immunity (1): Density (2); Cellular composition (3); Spatial localization; (4) Maturation status. Integrating these multidimensional parameters may facilitate the development of refined prognostic models and provide a framework for investigating treatment-response associations.

TLSs may therefore provide biologically informative features of the tumor immune microenvironment, but their clinical role should remain investigational until standardized assessment and prospective validation are available (80–83). Future models could integrate TLS pathology with molecular and spatial information, provided that incremental prognostic or predictive value is demonstrated.

### 5.4 TLS versus TILs: complementary biomarkers for clinical immune assessment

Tumor-infiltrating lymphocytes (TILs) and tertiary lymphoid structures (TLSs) capture related but distinct aspects of antitumor immunity. Stromal TIL assessment primarily quantifies the extent of lymphocytic infiltration, whereas TLS assessment additionally considers spatial organization and maturation, including B- and T-cell compartmentalization, follicular dendritic-cell networks, germinal-center formation, and high endothelial venules (80, 81). TLSs should therefore be viewed as complementary indicators of organized local immunity rather than simply a more specific form of TILs.

For routine breast cancer pathology, TIL assessment currently has a clearer path to clinical implementation because international recommendations have standardized stromal TIL scoring on H&E sections (80), and substantial evidence supports its prognostic value in TNBC (82). Exploratory analyses have also associated TIL levels with pathological complete response in patients receiving KEYNOTE-522-based neoadjuvant chemoimmunotherapy (83). More recent analyses of pembrolizumab-treated metastatic TNBC further support an association between higher TIL levels and clinical outcome (84). Nevertheless, TIL scoring mainly captures immune abundance and provides limited information about lymphocyte organization and maturation.

TLS assessment may provide additional biological information. Breast cancer studies have associated TLS abundance, localization, and maturation with prognosis and treatment response (70, 71), while multi-omics and spatial studies have described B-cell maturation, T-cell activation, and spatial immune programs that are not fully captured by overall lymphocyte density (74, 85). Mature TLSs have also been reported to retain prognostic significance after adjustment for TIL levels, and TLS-related transcriptional signatures have been associated with immune-checkpoint-inhibitor response (67). However, these observations do not establish TLSs as superior predictors of ICI benefit because much of the evidence remains retrospective or exploratory.

Accordingly, TILs and TLSs are best considered complementary rather than competing biomarkers. At present, stromal TIL assessment is more suitable for routine pathological immune evaluation because it has a standardized H&E-based scoring framework, whereas TLS should be regarded as an investigational complementary biomarker that may add spatial and maturation-related information. Neither TILs nor TLSs should currently be used alone to select patients for ICI therapy. Prospective head-to-head studies are needed to determine whether standardized TLS assessment provides incremental predictive value beyond TILs, PD-L1 status, tumor subtype, and other established clinicopathological variables; this is particularly important because TLS assessment remains sensitive to definition, staining strategy, maturation criteria, and specimen sampling.

## 6 Therapeutic potential of TLS remodeling in breast cancer

The translational relevance of TLSs can be considered in two broad areas: candidate biomarker applications and experimental strategies to induce or mature TLSs. The clinical associations of baseline TLS features are summarized in Section 5; here, the emphasis is on translational use and intervention. Both areas remain dependent on standardized assessment, prospective validation, and evidence of incremental clinical benefit (**Figure 5**). It should be noted that direct evidence for therapeutic TLS manipulation in breast cancer remains limited. Therefore, several strategies discussed below are derived from preclinical studies in other tumor types and are included as mechanistic or proof-of-concept evidence rather than as established therapeutic approaches in breast cancer.

**Figure 5:**
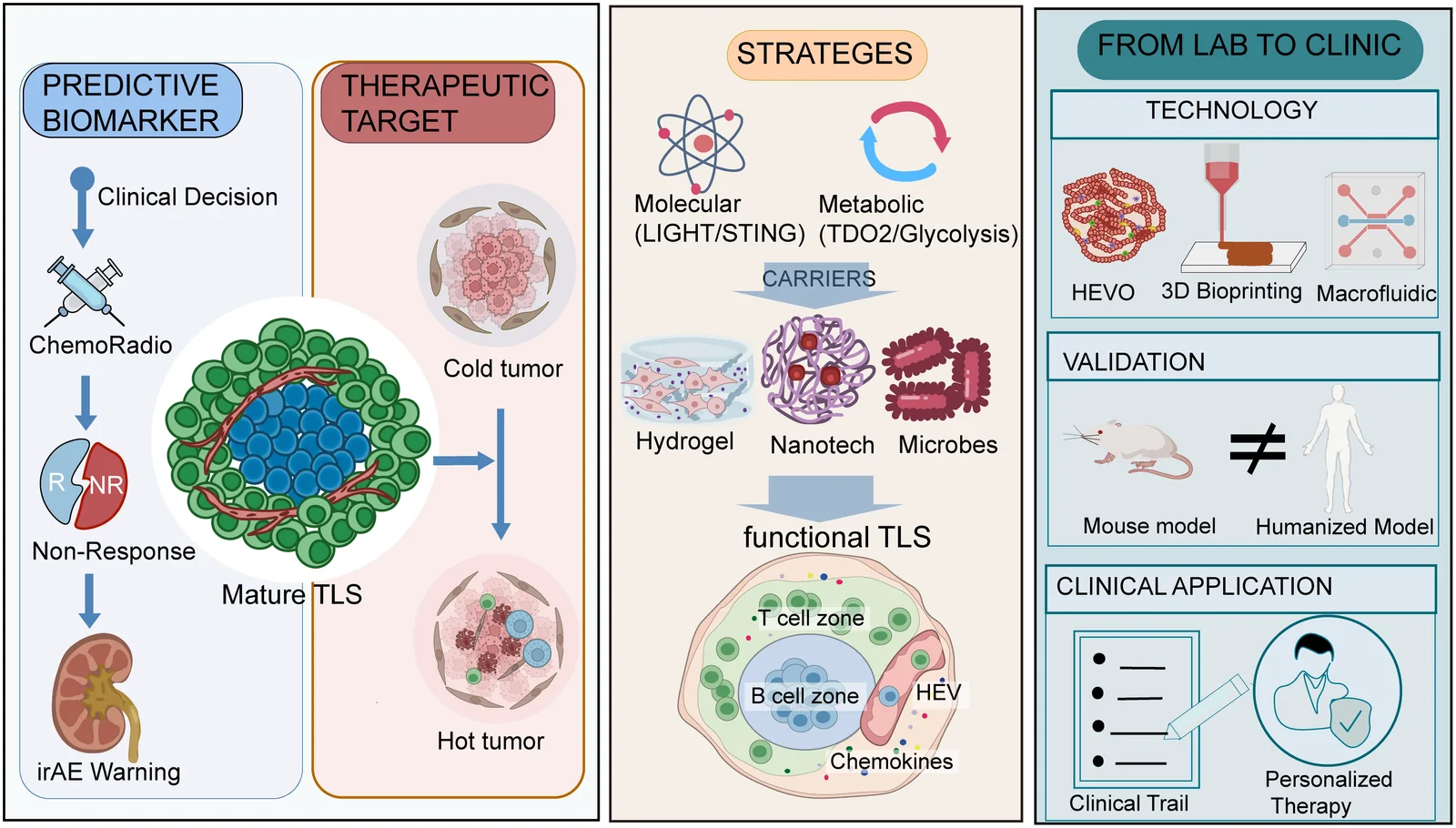
Clinical significance and therapeutic remodeling of Tertiary Lymphoid Structures (TLS). (Left) TLSs are being investigated as candidate biomarkers of treatment response and immune-related adverse events, as well as potential therapeutic targets for converting immune-cold tumors toward more immune-active states. (Center) Experimental TLS-promoting strategies include molecular and metabolic modulation together with biomaterial, nanotechnology, and microbial delivery systems designed to support functional TLS formation. (Right) Translation from laboratory models to clinical application requires enabling technologies, appropriate preclinical validation, clinical trials, and ultimately patient-specific treatment strategies.

### 6.1 TLSs as candidate biomarkers in precision oncology

The potential biomarker utility of TLSs spans baseline prognosis, treatment-response assessment, and treatment-associated immune toxicity. At present, however, these applications are investigational rather than established components of breast cancer clinical decision-making.

Longitudinal changes in TLS abundance, maturation, or cellular composition during therapy may reflect treatment-induced remodeling of the tumor immune microenvironment. In some studies, responders to TIL therapy or immune checkpoint blockade showed changes in memory B cells, dendritic-cell subsets, or TLS-related scores (75, 76). However, it remains unclear whether treatment-induced TLS changes provide independent prognostic or predictive information for recurrence, survival, or durable treatment benefit.

TLS-associated biology may also have context-dependent adverse effects. Although intratumoral TLSs are associated with favorable therapeutic responses in several settings, ectopic lymphoid aggregates and TLS-related programs have also been reported in some immune-related adverse events. For example, TLS-like structures have been described in ICI-associated acute interstitial nephritis, where activated B cells and plasma cells may contribute to pathogenic humoral responses (86).

Future TLS biomarker frameworks should therefore move beyond binary presence-or-absence scoring and consider anatomical location, maturation, cellular composition, and longitudinal change. If prospectively validated, such integrated models may improve biological stratification and characterization of treatment-associated immune effects, but their added value over established biomarkers must be demonstrated.

### 6.2 Preclinical strategies to induce or promote TLS maturation

#### 6.2.1 Molecular supplementation and immune activation

Supplementation or local delivery of lymphoid-organizing signals is one experimental strategy for promoting TLS-associated programs. LIGHT (TNFSF14), for example, signals through LTβR to induce chemokine expression such as CCL19, CCL21, and CXCL13 and can influence HEV-associated vascular programs. Preclinical intratumoral delivery approaches have reported increased lymphoid organization and antitumor immune activity (87). These findings provide proof of concept but do not establish efficacy or safety in breast cancer patients.

Innate immune activation represents another preclinical approach. STING agonists such as ADU-S100 can activate STING-TBK1-IRF3 signaling in dendritic cells and induce chemokine, lymphotoxin, and vascular programs associated with non-canonical TLS formation (88, 89). LTβR agonism can likewise promote HEV formation and lymphocyte recruitment in experimental models (90). Together, these studies provide a rationale for investigating STING- and LTβR-directed combinations; however, optimal dosing, toxicity, tumor specificity, and the functional maturity of induced TLSs remain unresolved. Other innate immune circuits may also participate; for example, IL-33-activated ILC2s have promoted TLS formation in pancreatic cancer models (91).

#### 6.2.2 Targeting the metabolic microenvironment

Metabolic competition and suppressive metabolites may constrain TLS formation and immune-cell function. In breast cancer models, inhibition of the TDO2-kynurenine pathway with LM10 has been associated with M1-like macrophage polarization, TLS-maturation-associated features, and enhanced antitumor activity when combined with PD-1 blockade (92, 93). These data remain preclinical and require validation of mechanism, safety, and reproducibility.

Tumor glycolysis, lactate accumulation, hypoxia, and altered lipid metabolism can suppress lymphocyte or myeloid-cell function (94–97). Interventions that modify these pathways may indirectly create conditions more permissive to TLS assembly, but direct evidence that metabolic intervention can reliably generate functionally mature TLSs in breast cancer remains limited.

#### 6.2.3 Precision delivery systems based on advanced biomaterials

Direct cytokine administration is limited by short half-lives, diffusion-related off-target effects, and the possibility of generating structurally incomplete or dysfunctional lymphoid aggregates. Biomaterial-based delivery systems are therefore being explored to provide more localized and sustained exposure to TLS-organizing signals (87, 98).

Biocompatible materials can be engineered as local scaffolds or depots for cytokines, chemokines, and antigens. Collagen matrices and responsive hydrogels containing factors such as CCL19/21 have generated TLS-like lymphoid aggregates with germinal-center-associated features in experimental models (98–101). Other designs use charge-based or supramolecular interactions to localize and activate T and B cells (102, 103). The extent to which these structures reproduce the cellular diversity and functional maturation of naturally occurring human TLSs remains uncertain.

Nanodelivery systems provide another means of coordinating local delivery of immune-stimulatory components. Reported preclinical examples include nanovaccines, dendritic-cell-membrane-based formulations, extracellular-vesicle systems, and manganese- or STING-containing constructs that increase TLS-associated immune features in several tumor models (98, 104–106). Because these studies use different tumor types, materials, endpoints, and definitions of TLS, comparative efficacy and translational relevance cannot yet be established.

The TPDA-ViBT-COF platform combined with 660-nm laser treatment has also been reported to promote lymphocyte recruitment and TLS-like/iTLS structures with selected functional features in preclinical MC38 and 4MOSC1 tumor models (107). Although such systems illustrate the possibility of engineering a supportive immune niche, confirmation of mature germinal-center function, durability, and safety is required before clinical translation.

Engineered bacteria with tumor-targeting capacity have similarly been investigated as local delivery vehicles for immune-organizing factors such as LIGHT (108, 109). These approaches remain experimental, and their potential advantages must be weighed against biosafety, control of bacterial distribution, and the challenge of translating findings from preclinical models to patients.

### 6.3 Functional versus structural TLS induction

Although multiple experimental strategies can induce TLS-like aggregates in tumors, whether these structures fully recapitulate the biological function of naturally occurring human TLSs remains uncertain (106–109). Structural aggregation should not be equated with functional activation. In particular, induced TLSs should be evaluated for germinal-center activity, antigen presentation, B-cell affinity maturation, plasma-cell differentiation, T-cell support, and durability rather than by lymphocyte clustering or HEV formation alone.

This distinction is central to evaluating TLS-directed interventions. An increase in TLS number or HEV-like structures may be biologically interesting but does not by itself demonstrate clinical benefit. Functional validation and standardized endpoints are therefore necessary to determine whether induced TLSs generate durable, tumor-specific adaptive immunity rather than transient or potentially dysregulated lymphoid organization.

### 6.4 Current limitations of TLS-targeted therapeutic strategies

Despite encouraging preclinical findings, several limitations continue to hinder translation of TLS-directed interventions. Species differences are a major concern because murine lymphoid organization, tumor models, and treatment responses may not fully reproduce human TLS biology. Human pluripotent stem cell-derived organoid systems containing high endothelial venule-like structures provide one approach for modeling selected aspects of lymphoid organization *in vitro* (110) but these systems do not yet reproduce the full complexity of patient tumors.

Three-dimensional bioprinting and microfluidic tumor-on-a-chip platforms may provide additional experimental control over stromal organization, multicellular interactions, fluid dynamics, and mechanical cues (111–113). These systems can recapitulate selected structural and cellular features relevant to TLS organization, but they remain research models and require validation against human tumor tissue and clinical outcomes.

Additional barriers include the long-term biosafety and manufacturability of novel biomaterials, heterogeneity in how induced TLSs are defined, and uncertainty about whether experimental TLS-like structures achieve durable functional maturity. Future translational studies should therefore prioritize standardized functional criteria, reproducibility across models, safety, and prospective evidence of clinical benefit in breast cancer.

## 7 Conclusion

Multi-omics studies have expanded the view of tertiary lymphoid structures from morphologically defined lymphocyte aggregates to spatially organized immune niches with heterogeneous cellular composition and maturation states. Single-cell and spatial transcriptomic approaches have generated detailed maps of B-cell, T-cell, myeloid, stromal, and vascular programs associated with TLSs and have provided mechanistic hypotheses for local adaptive immune coordination. However, molecular or spatial associations should not be interpreted as evidence that all TLSs are functionally equivalent or that TLS assessment is clinically actionable.

The next phase of TLS research should prioritize standardization and validation. Harmonized pathological definitions, maturation criteria, spatial sampling strategies, and analytical pipelines are needed to improve cross-study reproducibility. AI-assisted image analysis and non-invasive or longitudinal biomarkers may support future monitoring, but their performance must be established in independent cohorts and compared with existing pathological and molecular markers.

TLS-directed therapeutic strategies are also at an early translational stage. Controlled induction or maturation of TLSs through chemokine modulation, immune agonists, biomaterials, or engineered delivery systems is supported mainly by preclinical evidence. Demonstrating mature germinal-center function, tumor-specific immune benefit, biosafety, and reproducibility in humans will be essential before such approaches can enter routine practice. Overall, TLSs are promising research biomarkers and therapeutic targets, but their clinical utility in breast cancer will depend on prospective validation, standardized assessment, and evidence that they add value beyond established biomarkers and treatment-selection frameworks (**Figure 6**).

**Figure 6:**
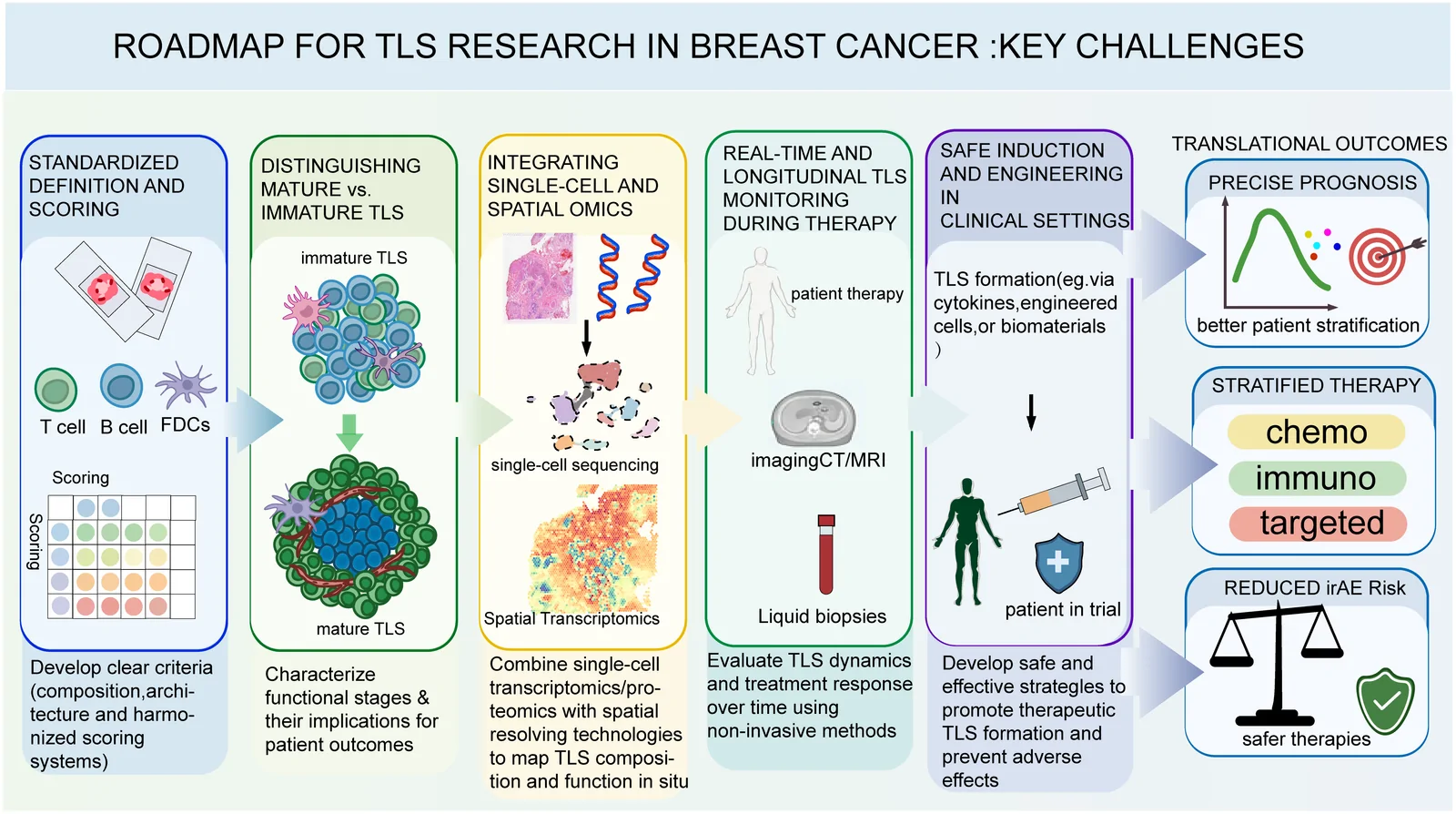
Challenges and future directions of tertiary lymphoid structure (TLS) research in breast cancer. (Left to right) Key priorities include standardized TLS definitions and scoring, reliable distinction between immature and mature TLSs, integration of single-cell and spatial multi-omics, longitudinal monitoring during therapy, and safe therapeutic induction or engineering of TLSs. (Right) Addressing these challenges may support more precise prognostic stratification, better-informed treatment selection, and safer therapeutic strategies.

## Glossary

TLSs: tertiary lymphoid structures
TILs: tumor-infiltrating lymphocytes
HER2: human epidermal growth factor receptor 2
H&E: hematoxylin and eosin
FDCs: follicular dendritic cells
PNAd: peripheral node addressin
E-TLSs: early tertiary lymphoid structures
PFL-TLSs: primary follicle-like tertiary lymphoid structures
SFL-TLSs: secondary follicle-like tertiary lymphoid structures
BCR: B-cell receptor
TLSNs: tertiary lymphoid structure networks
PD-1: programmed cell death protein 1
PD-L1: programmed death-ligand 1
STING: stimulator of interferon genes
TANs: tumor-associated neutrophils
AUC: area under the curve
DFS: disease-free survival
DRFS: distant recurrence-free survival
pCR: pathological complete response
ICB: immune checkpoint blockade
RECIST: Response Evaluation Criteria in Solid Tumors
TME: tumor microenvironment
BC: breast cancer
TNBC: triple-negative breast cancer
mIF: multiplex immunofluorescence
scRNA-seq: single-cell RNA sequencing
ST: spatial transcriptomics
SLOs: secondary lymphoid organs
MHC: major histocompatibility complex
DCs: dendritic cells
Tfh: follicular helper T cells
HEVs: high-endothelial venules
GCs: germinal centers
IHC: immunohistochemistry
LCM: laser capture microdissection
TAABs: tumor-associated atypical B cells
Trm: tissue-resident memory T cells
LTβ: lymphotoxin β
Tfr: follicular regulatory T cells
Tex: exhausted T cells
Texprog: progenitor exhausted T cells
Texterm: terminal exhausted T cells
CAFs: cancer-associated fibroblasts
FRC-like CAFs: fibroblastic reticular cell-like CAFs
TPCs: tumor pericytes
mTLS: mature TLS
ADCP: antibody-dependent cellular phagocytosis
apMCs: antigen-presenting mast cells
PMN-MDSCs: polymorphonuclear myeloid-derived suppressor cells
NAC: neoadjuvant chemotherapy
PFS: progression free survival
OS: overall survival
irAE: immune-related adverse events
LTβR: lymphotoxin beta receptor
Tregs: regulatory T cells
iTLS: inducible tertiary lymphoid structures
hPSCs: human pluripotent stem cells
HEVO: high endothelial venule organoids
IFN: interferon
ICIs: immune checkpoint inhibitors
qRT-PCR: quantitative reverse transcription polymerase chain reaction
cDC1s: conventional type 1 dendritic cells
TCR: T-cell receptor
NK cells: natural killer cells
ILC2s: group 2 innate lymphoid cells
MDSCs: myeloid-derived suppressor cells
HCC: hepatocellular carcinoma
CAR-T: chimeric antigen receptor T-cell
RCTD: Robust Cell Type Decomposition.
